# The Vineyard Yeast Microbiome, a Mixed Model Microbial Map

**DOI:** 10.1371/journal.pone.0052609

**Published:** 2012-12-26

**Authors:** Mathabatha Evodia Setati, Daniel Jacobson, Ursula-Claire Andong, Florian Bauer

**Affiliations:** Institute for Wine Biotechnology, Stellenbosch University, Stellenbosch, Western Cape, South Africa; Argonne National Laboratory, United States of America

## Abstract

Vineyards harbour a wide variety of microorganisms that play a pivotal role in pre- and post-harvest grape quality and will contribute significantly to the final aromatic properties of wine. The aim of the current study was to investigate the spatial distribution of microbial communities within and between individual vineyard management units. For the first time in such a study, we applied the Theory of Sampling (TOS) to sample gapes from adjacent and well established commercial vineyards within the same terroir unit and from several sampling points within each individual vineyard. Cultivation-based and molecular data sets were generated to capture the spatial heterogeneity in microbial populations within and between vineyards and analysed with novel mixed-model networks, which combine sample correlations and microbial community distribution probabilities. The data demonstrate that farming systems have a significant impact on fungal diversity but more importantly that there is significant species heterogeneity between samples in the same vineyard. Cultivation-based methods confirmed that while the same oxidative yeast species dominated in all vineyards, the least treated vineyard displayed significantly higher species richness, including many yeasts with biocontrol potential. The cultivatable yeast population was not fully representative of the more complex populations seen with molecular methods, and only the molecular data allowed discrimination amongst farming practices with multivariate and network analysis methods. Importantly, yeast species distribution is subject to significant intra-vineyard spatial fluctuations and the frequently reported heterogeneity of tank samples of grapes harvested from single vineyards at the same stage of ripeness might therefore, at least in part, be due to the differing microbiota in different sections of the vineyard.

## Introduction

Vineyards and grape berry surfaces provide a physical environment on which complex microbial communities comprising yeasts, bacteria and filamentous fungi establish themselves. In the wine industry, the species composition of these communities is of significant importance since the microbial species that are present on the berry may contribute to the fermentative process and therefore the aromatic properties of the resulting wine. This is of particular relevance in cases where the oenological practice includes spontaneous fermentations, as is the case in many wineries.

Data indicate that yeast populations on wine grapes increase from 10^2^–10^3^ cfu/g on immature berries to 10^3^–10^6^ cfu/g on mature berries. Yeast are spatially distributed over the grape berries and grape bunches, and also display temporal fluctuations in diversity over the course of grape berry development [1]–[4]. Species present on intact undamaged berries after véraison and until full ripeness have been reported to mainly belong to the group of oxidative basidiomycetous yeasts such as *Cryptococcus* spp., *Rhodotorula* spp., S*porobolomyces* spp., and *Filobasidium* spp., as well as to the dimorphic ascomycetous black yeast, *Aureobasidium pullulans* [1], [4], [5]. In the vineyard environment, these yeasts are typically associated with the phyllosphere, grapes and soil [1]. The oxidative ascomycetous yeasts (e.g. *Candida* spp., *Pichia* spp., and *Metschnikowia* spp.), and the fermentative ascomycetous yeasts (e.g. *Hanseniaspora/Kloeckera* spp.) have been found to be present at low concentrations on undamaged berries and appear often localized in those areas of the grape surface where some juice might escape [6], [7]. The incidence of these yeasts on damaged grapes increases rapidly and 10 fold increases have been reported [5], [7]. In contrast, the most relevant fermentative wine yeast, *Saccharomyces cerevisiae* only occurs at concentrations of less than 10–100 cfu/g berry [8].

The density and diversity of the grape microbiota may be influenced by many factors including climatic conditions, diseases, insect pests and viticultural practices [9]–[11]. Recently, differences in yeast populations associated with grapes obtained from organic and conventional farms have been reported [12]–[14], thus alluding to the possible impact of farming methods on grape microbiota. However, in these studies microbial diversity was only analysed after grapes were crushed and blended, thus using the juice as auto-enrichment, and either after 70 g/L of sugar was consumed or in the middle and end of alcoholic fermentation, when many species have been eliminated due to the high alcohol content. Such a strategy will have led to a significant enrichment of some species, and the elimination of many other species that were initially present on the grape. Furthermore, such an approach precludes a statistical validation of inter- and intra-vineyard variability.

In South Africa, wine grapes are produced using a range of farming methods from conventional to biodynamic farming. The majority of grapes are produced through what can be described as an intermediate scheme, the Integrated Production of Wine (IPW), which was established by the South African wine industry in 1998 [15]. This scheme embraces a more environmentally friendly farming system, including careful monitoring and understanding of diseases resulting in reduced input of biocides in the vineyard when compared with conventional farming [16]. The system also promotes the use of hay mulches and oats cover crops to improve soil moisture and fertility, as well as bait, ducks and other biocontrol strategies for pest control. However, integrated farming systems are not fully codified into rules, and do not have a regulated certification system [16]. In contrast, biodynamic farming is a specialised type of organic farming which prohibits any use of chemical fertilizers and pesticides as stipulated under the Demeter regulations [17]. In addition, biodynamic farming includes the use of specific fermented herbal and mineral preparations as compost additives and field sprays which are applied into the soil in animal organs e.g. bladder and cow horn [18].

Organic and biodynamic farming systems have been shown to enhance soil fertility and increase biodiversity [19]–[21]. In wheat plantations, microbial diversity has been found to be highest in biodynamic areas, followed by organically farmed and finally conventional plantations [19]. Although organic and biodynamic systems are globally becoming of increasing economic interest to wine producers, their impact on general vineyard health and wine quality has been the subject of relatively few studies. In particular, the impact of these practices on the vineyard ecosystem (including microbial diversity) is poorly understood.

The current study was aimed at evaluating microbial diversity associated with grapes obtained from conventional, biodynamic and integrated pest management vineyards, with a focus on epiphytic yeasts. The study also appears to be the first to assess intra-vineyard variability of microbial diversity. The data confirm previous results (on other crops) that biodynamic farming leads to a higher microbial diversity. It also shows that this diversity is unevenly distributed within individual vineyards, thus highlighting the importance of sampling multiple locations in the vineyard to assess the biodiversity of the ecosystem. From a wine making perspective, the data suggest that spatial fluctuations in microbial diversity might have a significant impact on downstream processes and analyses.

## Materials and Methods

### Vineyard Locations and Treatments

Cabernet sauvignon grape samples were collected from three directly adjacent vineyards. The vineyards, located in the Polkadraai region in Stellenbosch, South Africa (Fig. S1), were carefully selected to allow conclusive assessment of the impact of farming practices on both intra- and inter-vineyard microbial biodiversity. In particular, the vineyards are positioned on the same slope and aspect, and were all established in the same period (1994 and 1995). All vineyards also use the same trellising system (Perold 4 wire), row width (2.5 m) and vine interspacing width (1.4 m). However, each vineyard has been managed consistently and over a long period through strongly divergent farming methods, referred to as “conventional” (33° 57′41.50′′ S, 18° 45′11.87′′ E elev 179 m), “Integrated production” (33°57′40.65′′ S 18° 45′08.23′′ E elev 184 m) and “biodynamic” (33°57′39.33′′ S 18° 45′13.46′′ E elev 183 m). The conventional and biodynamic vineyard had the same cabernet sauvignon rootstock (R101-14) while the integrated vineyard has rootstock R110-CS23A. Management practices were as follows (see Table S1 for details): The “biodynamic” vineyard, was converted to “biodynamic” farming principles in 2000, and certified by Demeter International in 2006. The vineyard was treated regularly with Kumulus (sulphur), nordox (copper oxide), striker (organic fungicide with chitosan) and lime for the protection of powdery mildew and downy mildew, from leaf-fall until full bloom. The “integrated production” vineyard has been managed through the integrated pest and vineyard management system since its inception, which includes the use of chicken manure, inoculation of mycorrhizae and *Trichoderma* spp. into the soil, as well as the use of oats as cover crops. Pest management consisted of a combination of fungicides including hyperphos (mono- and dipotassium hydrogen phosphate), dithane (ethylene bisdithiocarbamate), Kumulus (80% sulphur), acrobat MZ (dimethomorph/mancozeb), talendo (proquinazid), curzate (cymoxanil/mancozeb) and stroby (kresoximethyl); and insecticides such as vantex (pyrethroid) and delmathrin, based on recommendations from an annual evaluation of the vineyard as per IPW guidelines. In contrast, the vines in the conventional vineyard were treated with chemical fertilizers applied when necessary and the vines were consistently treated with a combination of fungicides including folpan (N-(trichloromethyl)thio) phthalimide, rootex (phosphorous acid), cumulus, dithane, acrobat, talendo, cungfu (copper hydroxide) and topaz (mono- and di-potassium salts of phosphorous acid), and different stages from leaf-fall to full bloom (Table S1). Sprays 1, 2 and 3 were applied with designer, a non-ionic sticker to improve the spread, coverage and retention of the fungicides and insecticides. No specific permits were required for the described field studies as they do not form part of protected land or conservation areas, and have not been reported to contain any endangered species. The three vineyards are privately owned commercial entities consequently, permission to use them as a study site and to sample the grapes was granted independently by each of the owners.

### Sampling Design

According to the Theory of Sampling (TOS) [22]–[24], the most efficient manner to sample a two-dimensional lot is to linearise it (aka to ‘unfold’ or to ‘vectorize’ it), into an elongated one-dimensional lot from which to extract increments at equidistant intervals [25], [26]. This approach is optimal with respect to capturing and characterizing the heterogeneity present within the lot, offering a way to derive a minimum number of increments needed (Q) if based variographic analysis [27]; alternatively the number Q may reflect local logistical and/or economic constraints. From a sampling design perspective a vineyard block can be likened to a two-dimensional lot, where rows are easily unfolded into continuous series, in which panels (each containing 6 vines) make up a ‘group’. At each vine location, the increments were defined to equal bunches. In the present study, one increment (bunch) was collected from each group, with groups regularly spaced throughout the unfolded linear lot. Thus in the conventional vineyard six rows (no.s 9, 11, 13, 15, 17 and 19) were sampled, where bunches were collected between panel 3, 7 and 11. In the biodynamic vineyard seven rows (no.s 1, 4, 7, 10, 13, 16, 19) were sampled while in the integrated vineyard only three rows were targeted (no.s 115, 117 and 119); here the bunches were collected from panels 1, 3, 5, 7, 9 and 11 respectively (Fig. S1). Grape bunches were placed in sterile bags and transported to the laboratory and processed within 1 hour after harvest.

### Pseudoreplicaton Test

In order to test for pseudoreplication effects the following approach was implemented in Perl. A Cartesian coordinate system was created for each of the three vineyards utilizing the fact that the row width is 2.5 meters and the panel width is 9 meters. Given this each sampling point can be described as a two point vector and the distance between two sampling points can be calculated as follows:
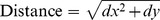
Where *dx* is the difference between the *x* coordinates and *dy* is the difference between the y coordinates. For each possible pair of sampling points within each vineyard the Pearson correlation of species detected via ARISA analysis was plotted against the distance between the sampling points and the R^2^ value calculated for each plot.

### Yeast Enumeration and Isolation

Thirty undamaged berries were collected from each bunch of grapes by using scissors cleaned with 70% ethanol and placed in 250 ml sterile pre-weighed Erlenmeyer flasks. The berries were then washed with 50 ml of saline solution comprising 0.9% w/v NaCl and 0.2% (v/v) Tween 80 to release the microorganisms [3]. This step was carried out at 30°C for 3 h with agitation on an Innova 5000 Gyrotory tier shaker (New Brunswick Scientific, Edison, New Jersey, USA) at 170 rpm. The washing solution was placed in 50 ml centrifuge tubes, followed by a centrifugation step at 5630× *g* for 10 min. The pellet was re-suspended in 10 ml fresh solution and used for yeast enumeration and community profiling using automated ribosomal intergenic spacer analysis (ARISA). For yeast isolation and enumeration, decimal dilutions (10^−1^ to 10^−3^) were prepared from the wash solutions, and 100 µl samples of each dilution were spread-plated in duplicate on Wallerstein nutrient agar (Sigma-Aldrich) supplemented with 34 mg/L chloramphenicol (Sigma-Aldrich) and 150 mg/L biphenyl (Riedel-deHaën, Seelze, Germany) to inhibit bacterial and mould growth, respectively. The plates were incubated at 30°C and examined daily for growth until the colonies were easily distinguishable. Where possible, 4–6 representatives of each colony-morphology were isolated from plates with ≤250 colonies and purified through two rounds of streak plating onto fresh agar plates. In addition, unique but infrequent colonies that were observed on plates with >250 colonies were also isolated. The isolates were maintained in 20% (v/v) glycerol at −80°C.

### DNA Extraction and ARISA Fingerprinting

The yeast communities associated with grapes were analyzed using PCR and ARISA. The remaining wash solutions were centrifuged at 5630× *g* for 10 min to collect microbial biomass. The pellet was re-suspended in lysis buffer and DNA was extracted as previously described by Hoffman [28]. The ITS1-5.8S-ITS2 rRNA region was amplified with the FAM labelled ITS1 primer (5′-TCCGTAGGTGAACCTGCGG-3′) and ITS4 (5′-TCCTCCGCTTATTGATATGC-3′), using the Phire® Plant Direct PCR kit (FINNZYMES OY, Espoo, Finland) under the following conditions: an initial denaturation of 6 min at 98°C, followed by 40 cycles of 98°C for 20 s, 54°C for 30 s, 72°C 1 min, and a final extension of 10 min at 72°C. The ARISA-PCR fragments were separated by capillary electrophoresis on an ABI3010xl Genetic Analyzer (Applied Biosystems, CA, USA) to obtain electropherograms of the different fragment lengths and fluorescent intensities. A ROX1.1 size standard was used [29]. The ARISA data was analysed using Genemapper 4.1 software (Applied Biosystems). A threshold of 50 fluorescent units was used to exclude background fluorescence. The software converted the fluorescence data into electropherograms, where the peaks represent fragments of different sizes, and the peak areas represent the relative proportion of these fragments. The number of peaks in each electropherogram was interpreted as the OTU richness in the community. The fragment lengths and fluorescence for each sample were aligned using an Excel Macro. Only fragment sizes larger than 0.5% of the total fluorescence and between 300 and 1000 bp in length were considered for analysis. A bin size of 3 bp for fragments below 700 bp and 5 bp for fragments above 700 bp was employed to minimize the inaccuracies in the ARISA profiles [30]. All elution points in the electropherograms that did not contain a peak in at least one sample were removed with the use of a custom built Perl program. This process resulted in a matrix in which each row represented a sample and each column represented an OTU (species). Principal component analysis (PCA) of the ARISA profile matrix was performed in STATISTICA software Version 10 [31].

### Vineyard Sampling Point Networks

An all-against-all comparison was done calculating the Pearson correlation between each and every sample vector in the ARISA matrix. As such, one is able to determine the correlation in population structure within and across vineyards. The relationships between samples were represented as a mathematical graph in order to form a correlation network with the nodes representing sampling point locations in each vineyard and the edges weighted with the Pearson correlations between the sampling point vectors. In order to select the highest correlations between sampling points a maximum spanning tree was created by transforming the edge weights into inverse correlations (by taking the difference between the number 1 and the absolute correlation values) and the subsequent use of a minimum spanning tree (mst) algorithm [32] on this inverse correlation network. A minimum spanning tree represents the shortest possible path through a graph and, as such, selects for the smallest inverse correlation (i.e highest correlation) pairs between all nodes in the network. The nodes were annotated with colours based on the vineyards the samples were taken from and the edge widths scaled with respect to the level of the original correlation values between the samples. The resulting network was visualized in Cytoscape [32].

### OTU Probability Networks

A probability matrix was created by dividing each element of a sample vector by the sum of all of the elements in the vector. Thus each resulting element represented the probability of that sample containing that particular OTU. A probability network was then created by creating edges between each sample and the OTUs for which there was a probability value >0 were used as edge weights. The sample nodes were annotated with colours based on the vineyards the samples were taken from. An edge-based spring embedded layout algorithm was applied to the resulting network which was then visualized in Cytoscape [32].

### Mixed-model Networks: Combining Correlation and Probability Networks

In order to represent the most probable microbial community structure of each sampling point a mixed-model network was developed as follows. The edge weights of the Vineyard Sampling point maximum spanning tree network described above were multiplied by 10 and the resulting re-weighted network Unioned with the probability network described above.

In order to select the highest probability edges between sampling points and OTUs a maximum spanning tree was created by transforming the edge weights into inverse values (by calculating the absolute difference between the number 1 and the edge weights) and the subsequent use of a minimum spanning tree (mst) algorithm [33] on this inverse edge-weighted network. After the mst algorithm was applied the original weights from both the correlation and probability networks were used as edge weights of the surviving edges. Edge thicknesses were then scale with respect to the edge weights and sampling point node sizes were scale with regard to degree (i.e. the number of edges incident to a node). OTU node sizes were scaled with regard to the probability of them occurring in the sample that they shared and edge with. The resulting network was then visualized in Cytoscape [32].

### Molecular Yeast Identification

Selected colonies were picked from the plate by using a sterile inoculating loop and DNA was extracted from the colonies using the protocol for rapid isolation of yeast DNA [28]. The isolates were then identified by amplifying the ITS1-5.8S-ITS2 rRNA region using the ITS1 and ITS4. PCR amplifications were carried out in a final volume of 25 µl containing 0.25 µM of each primer, 1× PCR reaction buffer, 1 mM MgCl_2_, 200 mM dNTPs, 1U Takara Ex Taq™ DNA polymerase (TaKaRa Bio Inc., Olsu, Shiga, Japan), 100 ng of DNA and sterilized de-ionized H_2_O. The PCR reaction was carried out using the following conditions: initial denaturation at 94°C for 2 min; 35 cycles of denaturing at 94°C for 30 s; annealing at 54°C for 45 s; an extension at 72°C for 1 min; and a final extension step of 10 min at 72°C. The PCR products were analysed by agarose gel electrophoresis; purified using the Zymoclean™ Gel DNA recovery kit (Zymo Research Corporation, Irvine, CA, USA), following the manufacture’s instruction, and then sequenced. The sequences obtained were assembled using BioEdit [34], and compared with sequences available in GenBank database available at the National Centre for Biotechnology Information (NCBI) http://www.ncbi.nlm.nih.gov/genbank/index.html using the basic local alignment search tool (BLAST) algorithm [35]. Sequences which displayed 98–99% identity to previously published species available at NCBI were binned into the same species. Sequences obtained in this study were deposited in NCBI GenBank database under accession numbers: JQ993367– JQ993394.

### Statistical Analysis

Relative abundance of species was calculated as a proportion of a particular species in the samples based on colony counts and frequency of isolation. Species richness was assessed using the Menhinick’s index while species evenness was assessed using Pielou index [36]. Shannon diversity index was used to assess the level of diversity in the three vineyards [12].

## Results

### Quantitative Analysis of Grape-associated Yeast Communities

The impact of farming systems on yeast population density was evaluated by culture-dependent methods following a 3 h rinsing of sound grape berries obtained from the conventional, biodynamic and integrated vineyards. The total yeast populations were higher in the biodynamic and conventional vineyard than in the integrated vineyard (Fig. S2). The total yeast population ranged from 4–8×10^4^ CFU/g on all vineyards, and the enumeration of cultivable population revealed no significant differences between the farming systems (*P* = 0,225).

### Inter- and Intra-vineyard Variability of the Total Fungal Community

**Figure 1:**
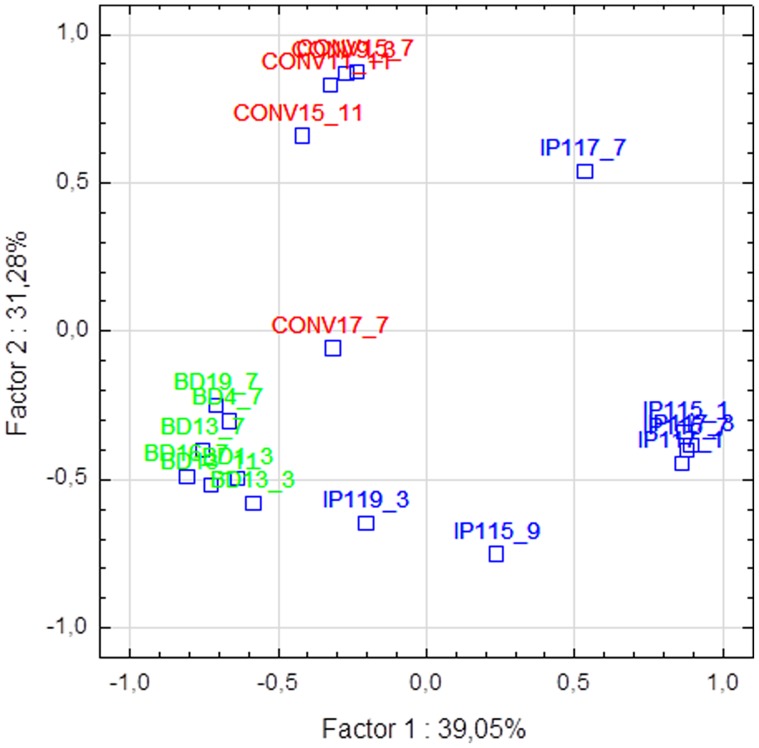
Principal component analysis based on fungal community structure assessed by ITS1-5.8S-ITS2 rRNA gene ARISA profiles. Biodynamic vineyard (Green), Conventional (Red), IPW (Blue).

**Figure 2:**
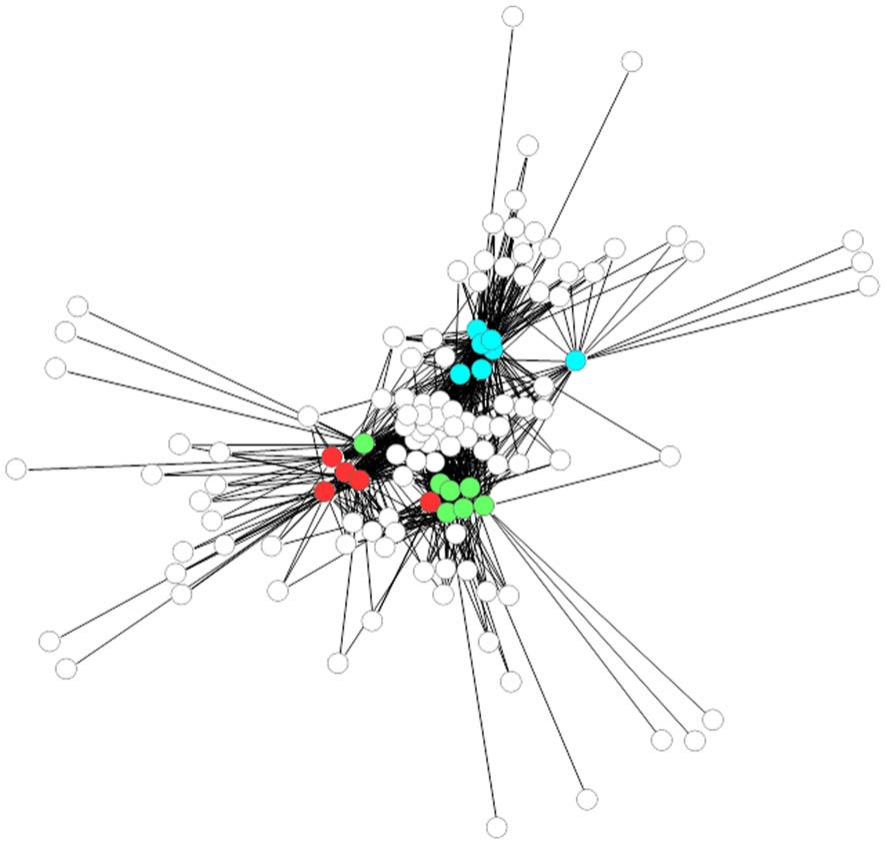
Probability network of OTU found at different sampling points. Sampling point nodes are coloured by farming practice: Biodynamic (Green), Conventional (Red) and IPW (Aqua). White nodes indicate OTUs common in the three vineyards.

**Figure 3:**
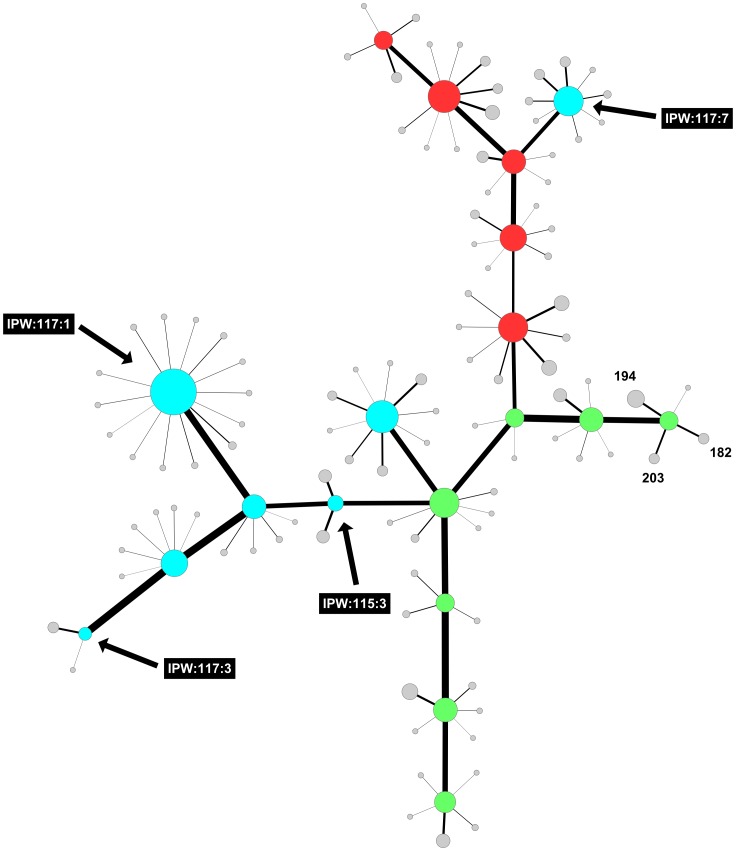
Mixed-Model Network: Sampling point Correlations and OTU probability distribution across samples. Sampling point nodes are coloured by farming practice: Biodynamic (Green), Conventional (Red) and IPW (Aqua). Sampling point node sizes are scale by degree and OTU nodes by the probability of occurring in the adjacent sampling point. White nodes represent OTUs most likely to be isolated from a given sampling point.

ARISA analysis was used to unravel fungal community structures associated with healthy/sound grapes in conventional, biodynamic and integrated pest management farming systems. Similar electropherograms were obtained from all the samples. Bands between 500 and 600 bp were dominant in all the vineyards, however, differences in fungal community structures were evident in the three vineyards. PCA analysis was performed on ARISA profiles to evaluate inter-vineyard variation. Each vineyard could be differentiated on the basis of the ARISA fingerprints (Fig. 1). The biodynamic and integrated vineyard could be separated on the first axis, with the integrated vineyard samples mainly clustered on the right hand side of the first factorial plane while the biodynamic vineyard samples clustered on the left hand side. In addition, the biodynamic and conventional vineyard could be further separated on the second axis which explained 31.2% of the total variance. The conventional vineyard samples mainly clustered in the top plane while the biodynamic vineyard samples were located in the lower factorial plane (Fig. 1). Community networks derived from the same ARISA data showed higher correlation between the biodynamic farming system and the integrated pest management system (Fig. S3). The community network of the three farming systems comprised highly connected OTUs revealing significant overlap between the three systems (Fig. 2), but also showing that there are several OTUs which are unique to specific farming systems. Once the link between microbial diversity and farming practices was established, we further explored intra-vineyard variability by evaluating the probability of certain OTUs being present in specific locations in the vineyard. The probabilistic species distribution patterns revealed interesting ecological patterns and for the first time confirmed intra-vineyard variability. For instance, in the integrated vineyard, row 117:panel 1 displayed a higher level of diversity, while row 115:panel 3 and row 117:panel 3, displayed the lowest diversity. Row 117:panel 7 comprised a unique fungal community which seemed more similar to the communities present in the conventional vineyard (Fig. 3). In contrast, in the biodynamic and conventional vineyard, the level of diversity within the rows and panels were similar, however, the OTUs represented at each site differed, such that the likelihood of isolating certain species from specific locations were variable. For instance, peaks 182 (518 bp), 194 (545 bp) and 203 (568 bp) are strongly associated with row 4:panel (7–8) in the biodynamic vineyard and therefore, the probability of isolating from this area is higher than with other sites. Similar observations were made for the conventional vineyard.

### Qualitative Diversity Analysis

**Figure 4:**
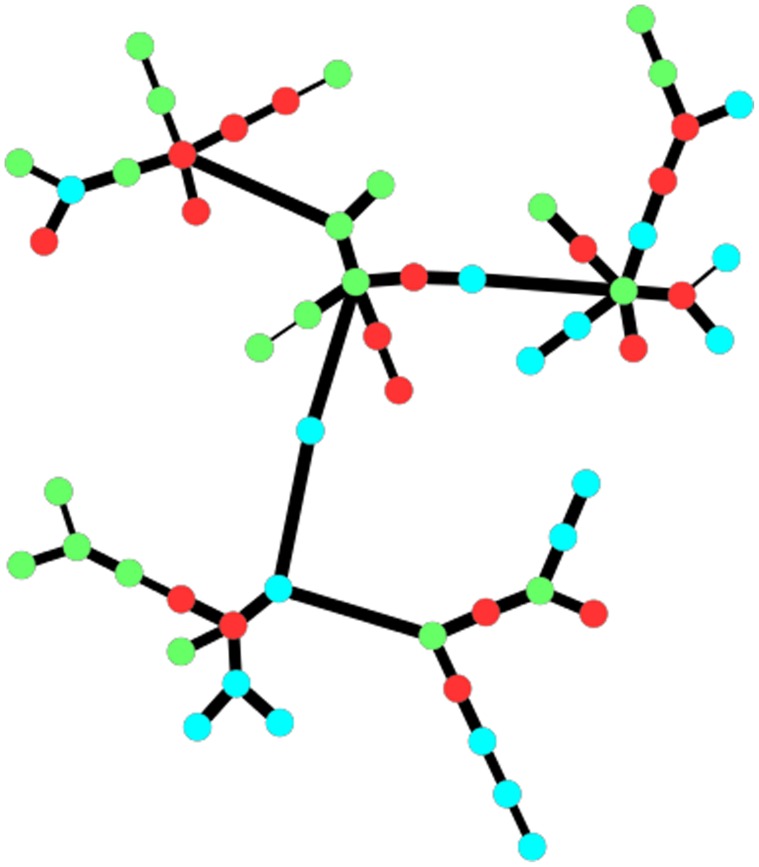
A correlation network of vineyard samples based on culturable yeast species. Nodes are coloured by farming practice: Biodynamic (Green), Conventional (Red) and IPW (Aqua).

A total of 628 yeast isolates from the three vineyards were analysed. Eleven species representing 8 genera were isolated from the conventional vineyard; the yeast isolated from the integrated vineyard represented 8 genera and 9 species, while 17 species representing 12 genera were isolated from the biodynamic vineyard. The biodynamic vineyard displayed a higher species richness and biodiversity than both the conventional and integrated vineyard (Table 1). Species evenness below 1 was found in all the vineyards. The dimorphic ascomycetous black yeast-like fungus, *Aureobasidium pullulans*, was widely distributed in the three vineyards (Table 2). *Cryptococcus* spp. were the second most prevalent yeast group with *Cr. magnus*, *Cr. carnescens* and *Cr. oeirensis* present in the three vineyards, while *Cr. laurentii* was only isolated from the integrated and biodynamic vineyard. The red pigmented yeasts including *Sporobolomyces roseus* and *Rhodotorula* spp., were also frequently isolated in the three vineyards. The biodynamic vineyard displayed some unique diversity of the minor yeast species including *Exophiala* sp., *Kazachstania* sp., *Sporisorium* sp., *Ustilago* sp. and *Meira* sp. (Table 2). However, these yeasts were not evenly distributed within the vineyard. For instance, *S. roseus*, *Ustilago* sp., *Kazachstania* sp. and *R. diobovatum* were isolated from three of the 21 sampling sites. In addition, only one sampling site contained 9 of the 17 species isolated from the biodynamic vineyard. In the conventional vineyard, only *Cr. magnus*, and *Cr. oeirensis* were widely distributed in the vineyard, while *Rh. sloofiae* and *S. roseus* were isolated from 4 of the 18 sampling sites. *Rh. glutinis* and *Cr. magnus* were present in 6 of the 18 sites in the integrated vineyard, while *Issatchenkia terricola* and *Cr. oeirensis* were only retrieved from 1 sampling site. A community correlation network generated from culturable yeast diversity does not result in any obvious partitioning of the three vineyards (Fig. 4).

**Table 1:** Ecological diversity indices determined using the yeast isolates obtained from the conventional (CONV), integrated (IPW) and biodynamic (BD) vineyard.

Vineyard	Menhinick’s index (Species richness)	Pielou’s index (Species evenness)	Shanon’s Diversity Index (Species diversity)
CONV	0.96	0.5	1.20
IPW	1.06	0.63	1.45
BD	1.45	0.76	2.15

**Table 2:** The occurrence and percentage distribution of yeasts associated with grape berries in the conventional (CONV), integrated (IPW) and biodynamic (BD) vineyards.

Species name	Accession number of closest relative	% Sequence identity	Yeast percentage distribution		
			CONV	IPW	BD
*Aureobasidium pullulans*	HM849057	99	70.4	63.2	52.5
*Cryptococcus magnus*	FN400937	100	9.2	7.9	6.6
*Cryptococcus carnescens*	EU149786	99	2.0	2.6	3.3
*Cryptococcus oeirensis*	AF444364	99	1.0	2.6	1.6
*Cryptococcus laurentii*	HM469461	100	–	2.6	2.5
*Cryptococcus flavescens*	FJ441026	100	–	–	3.3
*Rhodotorula slooffiae*	AF444589	99	4.1	–	4.1
*Sporobolomyces roseus*	AY015438	99	6.1	3.9	3.3
*Cryptococcus saitoi*	EU149781	99	–	–	0.8
*Rhodosporidium diobovatum*	HQ670682	99	–	–	5.7
*Kazachstania* sp.	AY582126	97	–	–	4.9
*Pichia caribbica*	EU568999	99	–	–	1.6
*Candida parapsilosis*	AB109228	99	–	–	1.6
*Meira geulakonigii*	GQ917051	99	–	–	1.6
*Exophiala* sp.	AB566310	97	–	–	1.6
*Sporisorium* sp.	AY344988	91	–	–	2.5
*Ustilago* sp.	AY740167	94	–	–	2.5
*Candida* sp.	FM178365	91	1.0	–	–
*Saccharomycete* sp.	FM178345	87	1.0	–	–
*Bullera dendrophila*	AF444443	94	3.1	–	–
*Rhodotorula glutinis*	HQ670677	99	–	7.9	–
*Cryptococcus randhawii*	AJ876528	97	–	2.6	–
*Issatchenkia terricola*	AY235808	99	1.0	6.6	–
*Rhodotorula nothofagi*	AY383749	99	1.0	–	–

### Testing for Potential Pseudoreplication

The three adjacent vineyards used in this study were as similar as possible with regard to macro-environmental factors as described in the Materials and Methods section. However, given that these were three different commercial vineyards which, as individual units, were managed with three different farming practices, the treatments, by definition, occurred within contiguous blocks. However, it is worth noting that the distances between sampling points within the Biodynamic and Conventional vineyards are often greater than the distance between the sampling points between those two vineyards. Nonetheless, pseudoreplication effects [37] from the sampling points within each vineyard are a possibility that we needed to address in order to ensure that environmental conditions [that have nothing to do with farming practice] present in each vineyard are not driving the selection of species. If this were the case one would expect to see a strong inverse correlation between the distance between sampling points and the species found in the samples. In order to test this, we devised a method which measures the distance between every possible sample pair within a vineyard and plots it against the species correlation values observed between those sample pairs. As can be seen in Figure S4, the R^2^ values for the conventional, biodynamic and IPW vineyards were 0.1063, −0.115 and 0.0106, respectively. As such, it appears that there is no correlative relationship between sample location within a vineyard and the species found there and thus apparently no pseudoreplication effect.

## Discussion

The current study evaluated the impact of farming systems *viz*. conventional, integrated and biodynamic viticultural practices on grape associated yeast diversity. Due to their ease of manipulation, grape berries are a good model fruit with which to easily capture the diversity. Our focus was on sound grape berries as they provide a better reflection of vineyard diversity since damaged berries may result in the isolation of some fermentative yeasts which have been shown to be harboured and disseminated by fruit flies e.g. *Drosophila* sp. [9].

The yeast counts obtained in the current study were in the same order of magnitude (10^4^–10^5^ cfu/g) as previous reports on the density of yeast populations on healthy/sound grape berries [3]–[5], [38]. Molecular ecological networks based on data obtained from ARISA analysis were used to discern inter- and intra-vineyard variability. Our experimental results demonstrated that there were significant node overlaps between the three farming systems which is probably due to generalist fungal populations which are commonly present in vineyard settings. This finding could also be corroborated with cultivation-based methods, which revealed that the three vineyards shared certain common yeast species such as *Aureobasidium pullulans*, *Cyptococcus magnus*, *Sporobolomyces roseus*, and *Rhodotorula glutinis*. The yeast-like fungus *A. pullulans* was found to be the dominant yeast inhabiting the grape berry surface. This observation is consistent with previous studies which have applied culture dependent methods as well as culture independent methods such as PCR-DGGE and FT-IR spectroscopy to monitor grape associated diversity [4], [6], [39]–[41]. In both culture-dependent and –independent approaches *A. pullulans* has been shown to account for 50–70% of the total population associated with undamaged grape berries. Other researchers have reported higher levels of this yeast-like fungus on organic vineyards than conventional vineyards. In contrast, our study shows a similar distribution of *A. pullulans* in the conventional, integrated and biodynamic vineyards. The dominance of this yeast-like fungus on grape surfaces has previously been attributed to its resistance to fungicides, the ability to detoxify CuSO_4_ and the ability to compete against other fungi [13], [40]. Our data further shows that despite the overlap between the three farming systems, there is sufficient difference in the total fungal community composition to separate the three farming systems from each other. These differences could mainly be due to minor yeast species. For instance, the biodynamic vineyard displayed unique biodiversity which comprised members of the genera *Sporisorium*, *Meira* and *Exophiala*, which have never previously been associated with the vineyard environment. A higher number of yeasts with biocontrol potential including *Rhodosporidium diobovatum*, *Meira geulakoningii*, and *Cryptococcus laurentii* were isolated from the biodynamic vineyard. *M. geulakoningii* is a mite-associated yeast which has been shown to be active against different species of mites e.g. carmine spider mite (*Tetranychus cinnabarinus*) and citrus rust mite (*Phyllocoptruta oleivora*) resulting in 100% mortality of the mites following treatment [42], [43]. This fungus could possibly be involved in suppressing mites such as *Tetranychus urticae* and *Colomerus vitis* which have been reported to be associated with grapevines in the Western Cape province of South Africa [44]. However, the distribution of this fungus and its actual role in the vineyard ecosystem needs to be investigated further. Other yeasts such as *Rh. diobovatum* and *Cr. laurentii* are also potential biocontrol agents against *B. cinerea*. This unique diversity could be due to the poor phytosanitary condition associated with the biodynamic vineyard, but it could also reflect the establishment of the natural enemies of different pests in the absence of pesticide application.

While previous research has alluded to spatial fluctuations within the vineyard, the extent to which such variations can occur with regard to grape berry associated microbiota has never been thoroughly investigated [1]. In addition to ruling out potential pseudoreplication effects from our sampling design, our data show that intra-vineyard variability can be attributed to considerable amounts of both inter- and intra-row spatial heterogeneity. This heterogeneity could be in part due to differences in immediate vine ecosystems and variation in inter-vine and intra-vine microclimates. For instance, the relative position of vines within the vineyard results in differences in the level of solar incoming radiation on the grape clusters, which in turn would affect the presence and proportion of pigmented yeasts such as member of the genera *Rhodotorula*, *Sporobolomyces* and *Rhodosporidium*. Ultimately, our study show that intravineyard variability is a significant factor, and may in some cases be higher than inter-vineyard differences even in cases of extreme treatment differences as applied to the blocks that were the subject of this study. This novel finding may lead to a reassessment of many previously published viticultural studies where the impact of vineyard treatments on wine composition was assessed. Indeed, this source of complexity has not been considered as a possible explanation for the observed heterogeneity of wines described in many such studies. Our data suggest that many differences may not derive from the differences in treatment, but rather differences in microbial diversity. The challenge in all field studies is the relatively large geo-spatial areas that need to be sampled and the logistical and financial limits to the number of samples that can be analysed. Often field studies take random samples from vineyards and, as such, may be reporting patterns that are not representative of the entire vineyard. The Theory of Sampling (TOS) has been developed over the past 50 years to deal with the sampling of large heterogeneous lots of material. From the results presented here it appears that viewing a vineyard as a large, heterogeneous two-dimensional lot, and using the TOS approach of lot-linearization and incremental sampling is an appropriate approach for such field studies in order to maximise the probability of collecting the most representative set of samples from a vineyard.

The current study shows unequivocally, that although culture-based methods generated interesting results regarding the microbial ecology of the vineyard, they were not an adequate approach to decipher the impact of farming systems on grape associated diversity probably due to the fact that this approach although not intentional, selects for certain groups of organisms, either due to their non-fastidious nature and rapid growth while excluding others whose cultivation requirements remain unknown. Overall, it was found that the sound grape berries are mainly colonized by oxidative yeasts, mainly *Aureobasidium pullulans* and *Cryptococcus* spp. These yeasts have been shown to occur on the surface of other parts of the phylloplane such as leaves and bark, as well as the soil [45]. Similar results have been reported in previous studies, and it is becoming more evident that although these yeasts are irrelevant to winemaking due their inability to ferment sugars or survive in wine, they represent the resident microbiota of grape berries [1], [2]. The biodynamic vineyard displayed higher diversity (*H’* = 2.15), while the conventional vineyard displayed the lowest diversity (*H’* = 1.20). However, the species evenness in the three vineyards was below 1, indicating the sparse distribution of the minor species. It could be speculated that the high diversity of the biodynamic vineyard is attributable to the fact that no fungicides except CuSO_4_ are applied on the vineyard, however this needs to be investigated. Several studies have shown that fungicides do not have an impact on *Cryptococcus* spp., *Rhodotorula* spp. and *A. pullulans*, which may explain their higher frequency on all the vineyards [6], [40]. However, the impact of fungicides on other yeasts has not yet been investigated. Isolates of the genus *Kazachstania* were also obtained from the biodynamic vineyard. Although not frequently encountered in vineyard settings, the genus *Kazachstania* its association with wine grapes has been previously demonstrated [46]. Given the close proximity of the three vineyards, it could be speculated that there is limited cross-transfer of yeasts from one vineyard to the other especially regarding the minor yeasts. However, an in-depth analysis using culture-independent methods e.g. metagenomics would be best suited to provide further insight. In addition, it would be important to evaluate the microbial diversity over several vintages and at different grape ripening stages to confirm whether the distinction between the vineyards is persistent.

## Supporting Information

Figure S1 **Geographic location of the study sites.** IPW = integrated production of wine; BD = biodynamic; CONV = conventional.
(TIF)

Figure S2 **Total yeast populations enumerated on grape berry surfaces from biodynamic (BD), integrated production (IPW) and conventional (CONV) vineyards.** The results were averaged from duplicate dilutions and are expressed as means ± SE of total samples. Error bars represent the standard error of means.
(TIF)

Figure S3 **Correlation Network of Microbial Populations at different Sampling Points.** Nodes are coloured by farming practice: Biodynamic (Green), Conventional (Red) and IPW (Aqua). Edge width is scaled to correlation value, so the thicker the edge the stronger the correlation.
(TIF)

Figure S4 **Species Correlation vs Spatial Distribution for each sample pair within vineyards for A) Conventional, B) Biodynamic and C) IPW vineyards.**
(TIF)

Table S1 **Spray programme for the biodynamic, conventional and integrated vineyard from leaf-fall till full bloom.**
(DOCX)
