# Correction: The Vineyard Yeast Microbiome, a Mixed Model Microbial Map

**DOI:** 10.1371/annotation/b9d307d9-f5c1-4e0d-8945-c5a747b6f58e

**Published:** 2013-09-19

**Authors:** Mathabatha Evodia Setati, Daniel Jacobson, Ursula-Claire Andong, Florian Franz Bauer

There was an error in the last author's name. The correct name is: Florian Franz Bauer.

The correct citation is: Setati ME, Jacobson D, Andong U-C, Bauer FF (2012) The Vineyard Yeast Microbiome, a Mixed Model Microbial Map. PLoS ONE 7(12): e52609. doi:10.1371/journal.pone.0052609

The correct Author Contributions are: Conceived and designed the experiments: MES DJ FFB. Performed the experiments: MES U-CA DJ. Analyzed the data: MES U-CA DJ. Contributed reagents/materials/analysis tools: MES DJ FFB. Wrote the paper: MES DJ FFB. 

