# Supplementary figures and images for: The Vineyard Yeast Microbiome, a Mixed Model Microbial Map
Source: PLoS One. 2012 Dec 26;7(12):e52609. doi: 10.1371/journal.pone.0052609 (PMC3530458; doi:10.1371/journal.pone.0052609)

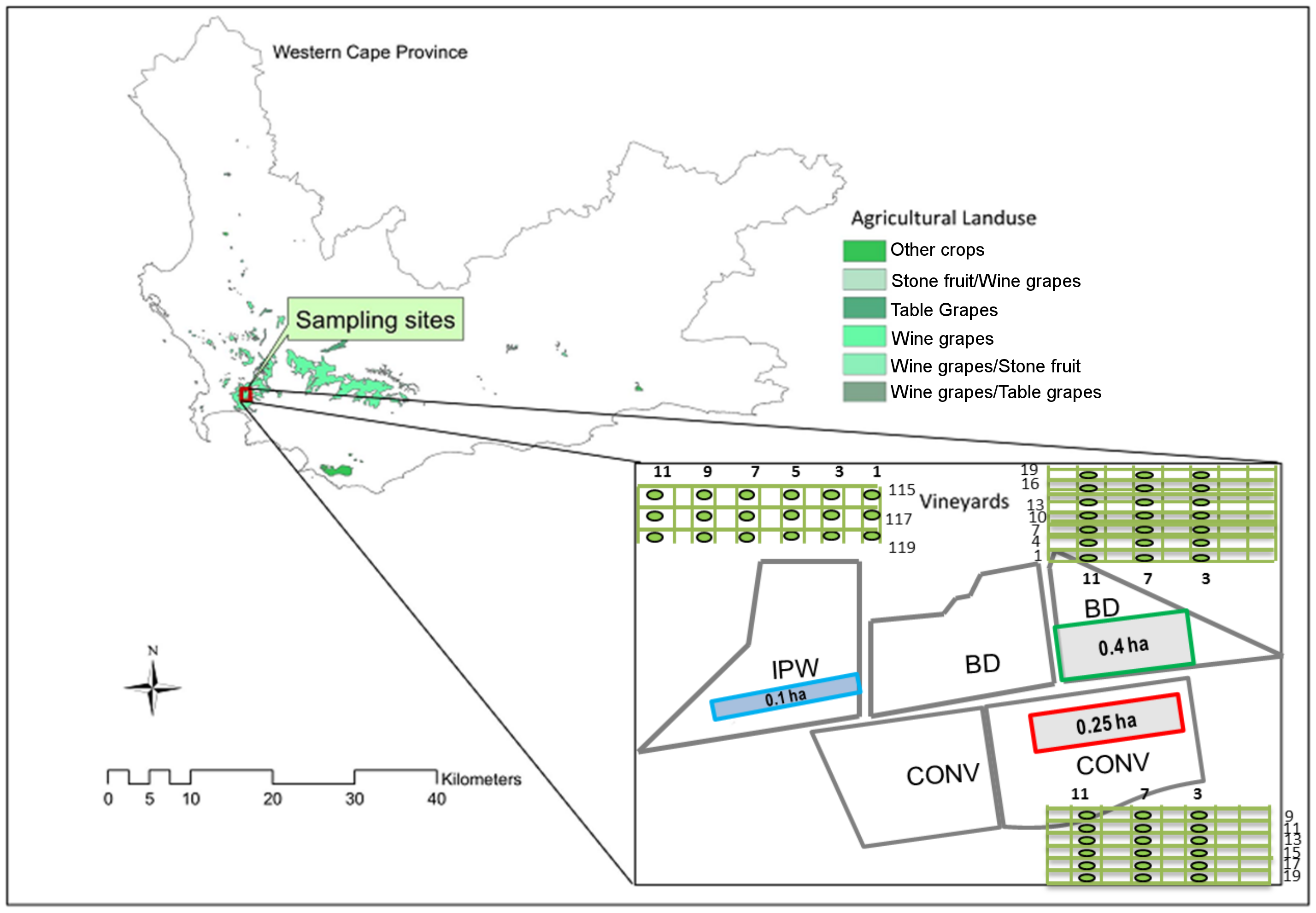

Supplement: Figure S1 — Geographic location of the study sites. IPW = integrated production of wine; BD = biodynamic; CONV = conventional. (TIF) [file pone.0052609.s001.tif]

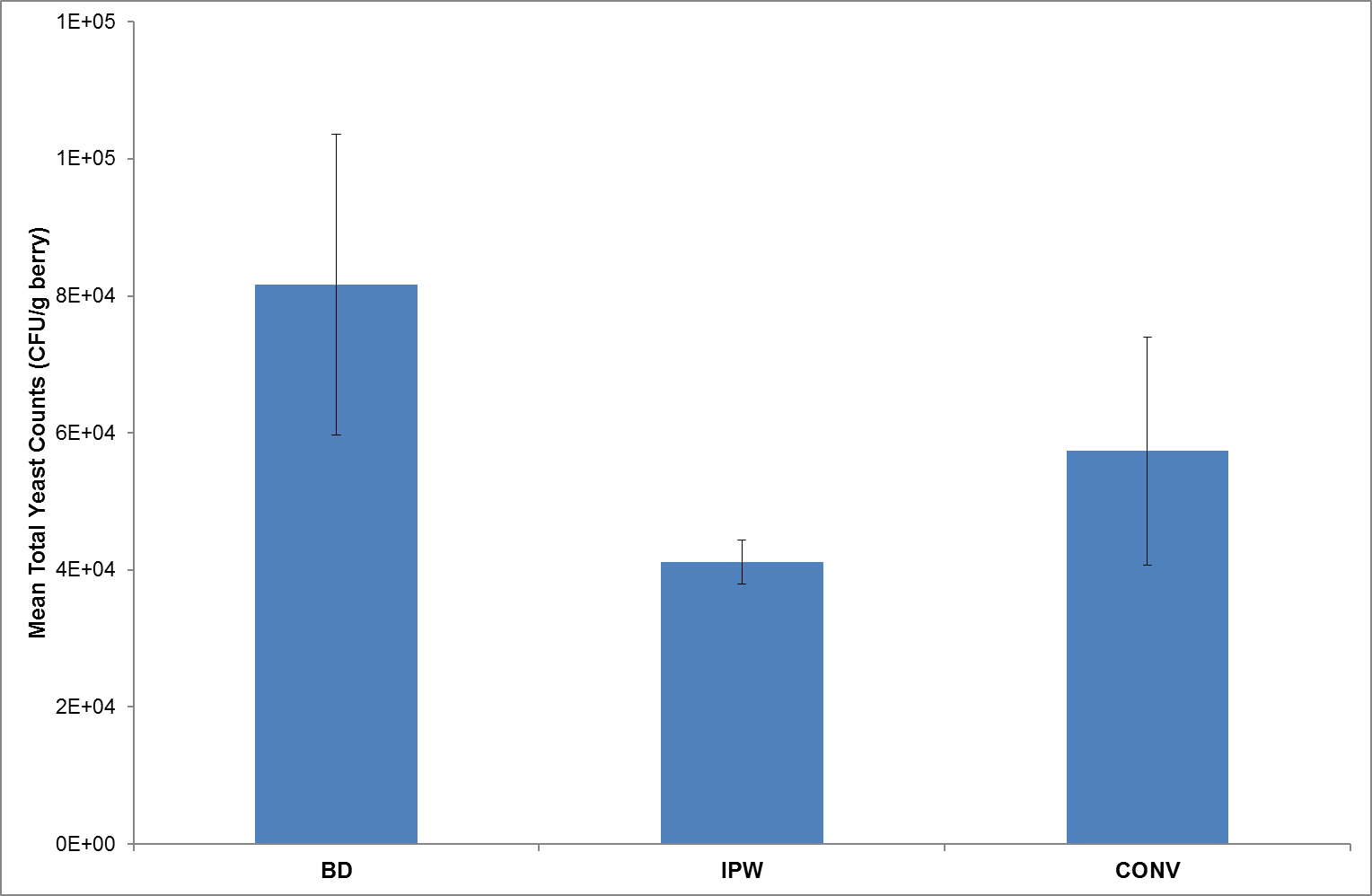

Supplement: Figure S2 — Total yeast populations enumerated on grape berry surfaces from biodynamic (BD), integrated production (IPW) and conventional (CONV) vineyards. The results were averaged from duplicate dilutions and are expressed as means ± SE of total samples. Error bars represent the standard error of means. (TIF) [file pone.0052609.s002.tif]

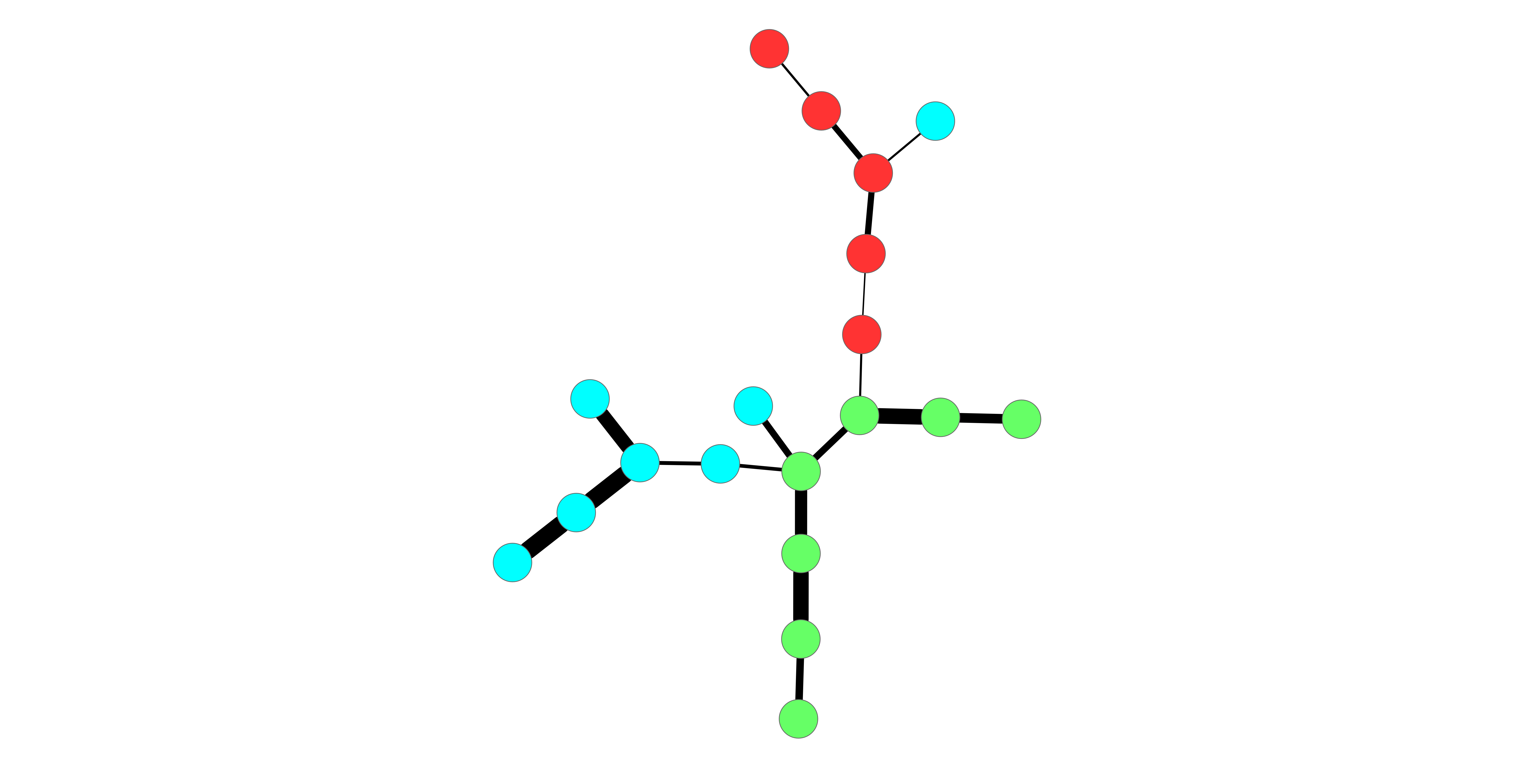

Supplement: Figure S3 — Correlation Network of Microbial Populations at different Sampling Points. Nodes are coloured by farming practice: Biodynamic (Green), Conventional (Red) and IPW (Aqua). Edge width is scaled to correlation value, so the thicker the edge the stronger the correlation. (TIF) [file pone.0052609.s003.tif]

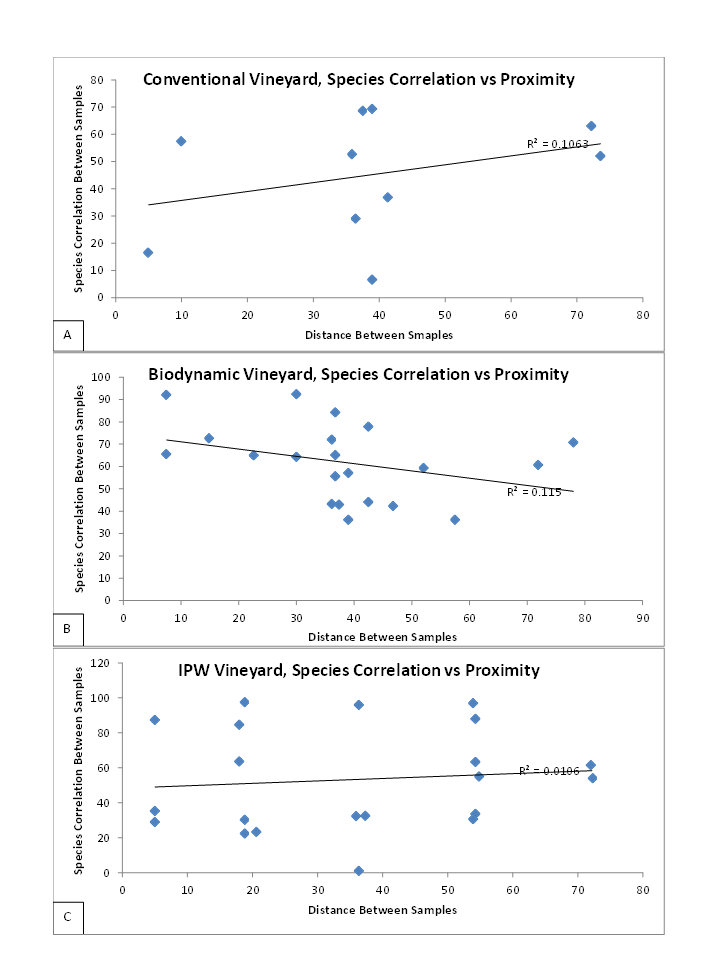

Supplement: Figure S4 — Species Correlation vs Spatial Distribution for each sample pair within vineyards for A) Conventional, B) Biodynamic and C) IPW vineyards. (TIF) [file pone.0052609.s004.tif]
