# Supplementary material for: The Vineyard Yeast Microbiome, a Mixed Model Microbial Map
Source: PLoS One. 2012 Dec 26;7(12):e52609. doi: 10.1371/journal.pone.0052609 (PMC3530458; doi:10.1371/journal.pone.0052609)
Supplement: Table S1 — Spray programme for the biodynamic, conventional and integrated vineyard from leaf-fall till full bloom. (DOCX) [file pone.0052609.s005.docx]

**Table S1.** Spray programme for the biodynamic, conventional and integrated vineyard from leaf-fall till full bloom

| **Spray** | **Biodynamic** | | **Conventional** | | **Integrated** | |
| --- | --- | --- | --- | --- | --- | --- |
|  | Product | Dosage (g/ha) | Product | Dosage | Product | Dosage |
| 1 | Nordox | 390 | Folpan | 312,5 g/ha | Hyperphos | 2 kg/ha |
|  | Striker | 450 | Kumulus | 2,5 Kg/ha | Dithane | 600 g/ha |
|  |  |  |  |  | Kumulus | 3 kg/ha |
|  |  |  |  |  | Acrobat | 1 kg /ha |
|  |  |  |  |  |  |  |
| 2 | Nordox | 390 | Folpan | 312,5 g/ha | Kumulus | 3 kg/ha |
|  | Kumulus | 600 | Kumulus | 2,5 Kg/ha | Acrobat | 1 kg/ha |
|  |  |  | Rootex | 750 ml/ha |  |  |
|  |  |  |  |  |  |  |
| 3 | Nordox | 225 | Dithane | 1 kg/ha | Kumulus | 3 kg/ha |
|  | Kumulus | 600 | Kumulus | 3 kg/ha | Acrobat | 1,5 kg/ha |
|  |  |  | Rootex | 750 ml/ha | Talendo | 1,5 kg/ha |
|  |  |  |  |  |  |  |
| 4 | Nordox | 390 | Acrobat | 1 kg/ha | Hyperphos | 3 kg/ha |
|  | Kumulus | 600 | Talendo | 25 g/ha | Curzate | 1,5 kg/ha |
|  |  |  |  |  |  |  |
| 5 | Nordox | 390 | Acrobat | 1,5 kg/ha | Talendo | 1,5 kg/ha |
|  | Kumulus | 600 | Talendo | 25 g/ha | Hyperphos | 4 kg/ha |
|  |  |  |  |  |  |  |
| 6 | Nordox | 390 | Cungfu | 605 g/ha | Curzate | 2 kg/ha |
|  | Kumulus | 600 | Kumulus | 3 kg/ha | Kumulus | 3 kg/ha |
|  | Lime | 500 |  |  |  |  |
|  |  |  |  |  |  |  |
| 7 | Nordox | 390 | Dithane | 2 kg/ha | Hyperphos | 4 kg/ha |
|  | Kumulus | 600 | Topaz | 30 g/ha | Dithane | 1,2 kg/ha |
|  | Lime | 500 |  |  | Stroby | 150 g/ha |
|  |  |  |  |  |  |  |
| 8 |  |  | Dithane | 2 kg/ha | Dithane | 1,2 kg/ha |
|  |  |  | Topaz | 30 g/ha | Stroby | 150 g/ha |
